# A One-Health Approach to Investigating an Outbreak of Alimentary Tick-Borne Encephalitis in a Non-endemic Area in France (Ain, Eastern France): A Longitudinal Serological Study in Livestock, Detection in Ticks, and the First Tick-Borne Encephalitis Virus Isolation and Molecular Characterisation

**DOI:** 10.3389/fmicb.2022.863725

**Published:** 2022-04-11

**Authors:** Gaëlle Gonzalez, Laure Bournez, Rayane Amaral Moraes, Dumarest Marine, Clémence Galon, Fabien Vorimore, Maxime Cochin, Antoine Nougairède, Catherine Hennechart-Collette, Sylvie Perelle, Isabelle Leparc-Goffart, Guillaume André Durand, Gilda Grard, Thomas Bénet, Nathalie Danjou, Martine Blanchin, Sandrine A. Lacour, Boué Franck, Guillaume Chenut, Catherine Mainguet, Catherine Simon, Laurence Brémont, Stephan Zientara, Sara Moutailler, Sandra Martin-Latil, Nolwenn M. Dheilly, Cécile Beck, Sylvie Lecollinet

**Affiliations:** ^1^ANSES, INRAE, Ecole Nationale Vétérinaire d’Alfort, UMR VIROLOGIE, Laboratoire de Santé Animale, Maisons-Alfort, France; ^2^ANSES, Nancy Laboratory for Rabies and Wildlife, Malzéville, France; ^3^ANSES, INRAE, Ecole Nationale Vétérinaire d’Alfort, UMR BIPAR, Laboratoire de Santé Animale, Maisons-Alfort, France; ^4^Bacterial Zoonosis Unit, Laboratory for Animal Health, ANSES Maisons-Alfort, Paris-Est University, Paris, France; ^5^Unité des Virus Émergents (UVE), Aix-Marseille Univ-IRD 190-Inserm 1207-IHU Méditerranée Infection, Marseille, France; ^6^ANSES Laboratory for Food Safety, Université Paris-Est, Maisons-Alfort, France; ^7^French National Reference Centre for Arbovirus, Armed Forces Biomedical Research Institute, Marseille, France; ^8^Santé Publique France, French Public Health Agency, Auvergne-Rhône-Alpes Regional Office, Lyon, France; ^9^Regional Health Agency (Agence Régionale de Santé), Auvergne-Rhône-Alpes, Lyon, France; ^10^Local Health Authority, Direction Départementale de la Protection de la Population de l’Ain, Bourg-en-Bresse, France

**Keywords:** tick-borne encephalitis virus, one health, alimentary route, outbreak, environmental investigation

## Abstract

Tick-borne encephalitis virus’ (TBEV) geographic range and the human incidence are increasing throughout Europe, putting a number of non-endemic regions and countries at risk of outbreaks. In spring 2020, there was an outbreak of tick-born encephalitis (TBE) in Ain, Eastern France, where the virus had never been detected before. All patients but one had consumed traditional unpasteurised raw goat cheese from a local producer. We conducted an investigation in the suspected farm using an integrative One Health approach. Our methodology included (i) the detection of virus in cheese and milk products, (ii) serological testing of all animals in the suspected farm and surrounding farms, (iii) an analysis of the landscape and localisation of wooded area, (iv) the capture of questing ticks and small mammals for virus detection and estimating enzootic hazard, and (v) virus isolation and genome sequencing. This approach allowed us to confirm the alimentary origin of the TBE outbreak and witness in real-time the seroconversion of recently exposed individuals and excretion of virus in goat milk. In addition, we identified a wooded focus area where and around which there is a risk of TBEV exposure. We provide the first TBEV isolate responsible for the first alimentary-transmitted TBE in France, obtained its full-length genome sequence, and found that it belongs to the European subtype of TBEV. TBEV is now a notifiable human disease in France, which should facilitate surveillance of its incidence and distribution throughout France.

## Introduction

Tick-borne encephalitis (TBE), caused by the tick-borne encephalitis virus (TBEV), is the most important tick-borne zoonotic disease in Europe and Asia from a medical perspective (Süss, 2011). Indeed, even though TBEV infection in humans often causes unspecific febrile symptoms and remain unnoticed, it can result in severe neurological diseases, including encephalitis, meningitis, and meningoencephalitis, with frequent incomplete recovery and, though rarely, death (Bogovic et al., 2010; Kohlmaier et al., 2021). TBEV is a positive-sense, single-stranded RNA virus of the genus *Flavivirus*, family *Flaviviridae* that circulates preferably among ticks of the genus *Ixodes* and small mammals, but large mammals and migrating birds also contribute to the virus geographic distribution and dispersion (Labuda et al., 1997; Randolph et al., 1999; Waldenström et al., 2007; Lindquist and Vapalahti, 2008; Gunnar et al., 2009; Jaenson et al., 2012; Tonteri et al., 2016; Paulsen et al., 2019). TBE most often results from tick bites, however, cases can also result from consumption of unpasteurised milk or dairy products from infected cows, goats, and ewes (Gresíková et al., 1975; Holzmann et al., 2009; Balogh et al., 2010; Cisak et al., 2010; Caini et al., 2012; Hudopisk et al., 2013; Markovinović et al., 2016; Brockmann et al., 2018; Dorko et al., 2018; Ilic et al., 2020; Chitimia-Dobler et al., 2021). Indeed, ruminants are also infected when bitten by an infected tick. Even if they remain asymptomatic, virus excretion in milk has been reported for cows, sheep, and goats (Gresíková et al., 1975; Balogh et al., 2010; Cisak et al., 2010; Caini et al., 2012). Pasteurisation prevents TBEV transmission through inactivation of virus infectivity (Rónai and Egyed, 2020). The increasing geographic range of TBEV and its patchy distribution around local foci puts an increasing number of countries at risk of sporadic occurrence of TBEV infections that can both be difficult to diagnose and prevent (Blaskovic, 1967; Deviatkin et al., 2020).

Tick-borne encephalitis virus (TBEV) is endemic throughout Central and Eastern Europe and has so far been detected in twenty-seven European countries. In the past decades, TBE human incidence has been increasing in several European countries (ECDC, 2012, 2018), with new foci detected in the United Kingdom (Holding et al., 2020) and Netherlands (Jahfari et al., 2017), expanding into previously unaffected areas (Daniel et al., 2004; Lukan et al., 2010; Donoso Mantke et al., 2011; Martello et al., 2014; Brabec et al., 2017; Rieille et al., 2017; Hellenbrand et al., 2019; Alfano et al., 2020). These observations, associated with evidence of a recent increase in TBEV diversity in Europe, suggest that TBEV may be regarded as an emerging disease (Deviatkin et al., 2020). France is located on the border of the known distribution of TBEV in Europe, and very few human cases are usually diagnosed annually, mainly in the Alsace region (about 10–30 cases per year, 0.5/100,000 inhabitants) based on suspect clinical presentation and the detection of TBEV-specific antibodies (Hansmann et al., 2006; Herpe et al., 2007; Donoso Mantke et al., 2011; Velay et al., 2018).

Herein, we report the first outbreak of alimentary TBEV in France and describe the investigation that was conducted within the suspected farm and surrounding area. In April 2020, an outbreak of encephalitis and meningoencephalitis occurred in the Ain department (Eastern France) where TBEV had never been detected. Consequently, a sanitary alert was decreed on May 10. Within a month, epidemiological investigation evidenced that 43 patients with encephalitis, meningoencephalitis, or flu-like symptoms, except for one, had consumed fresh goat cheese made of raw milk named “faisselle” coming from a single local producer. Immediately, the suspected goat flocks were confined into stall, unpasteurised goat cheese production was stopped, raw cheese produced before the alert was withdrawn from commercial markets, and an investigation was conducted within the farm. We confirmed the alimentary transmission by demonstrating the presence of TBEV in a batch of cheese and goat milk. We also monitored the serological status and virus excretion in milk of the animals after control measures had been established and demonstrated their efficacy since no TBEV infection has been reported later than early June. We assessed enzootic hazard for animal exposure through tick bites measured as the density of infected ticks in the pasture and in the neighbour woodland. The full-length virus genome sequence was obtained from milk, cheese, and tick samples, allowing the virus molecular characterisation, and an isolate was obtained. Finally, we assessed TBEV seroprevalence in surrounding farms to evaluate whether TBEV had been silently established in the area or not.

## Materials and Methods

**FIGURE 1:**
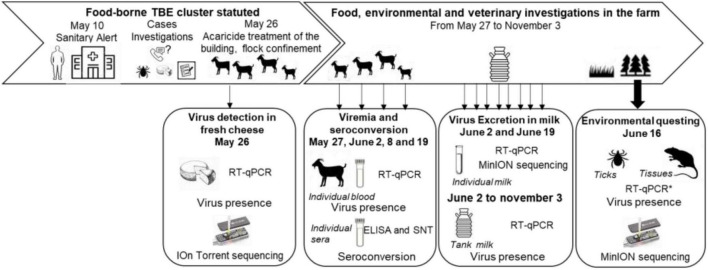
Timeline of the food, environmental and veterinary investigations, and sampling scheme from April to November 2020.

An overview of the timeline of sampling collections is provided in Figure 1.

### Cheese Sampling

Case investigations performed by the French public health agency pointed out that one case still had cheese from the suspected farm at home at the time of the investigation. This fresh cheese was produced on April 28. The Ain local authorities recalled cheese produced at the suspected farm. Five samples from the batch produced on April 28 and 74 from other batches were sent to the National Reference Centre (NRC) for Arboviruses for screening of the TBE genome. Confirmation was conducted at the Food-safety national reference laboratory, ANSES, Maisons-Alfort.

### Serum, Blood, Milk, and Tick Sampling

#### From Animals at the Suspected Farm

The farm suspected to be the source of the infection included 56 dairy goats, three dairy cows, and four suckling cows grazing on the same pasture.

The goats had grazed in only one pasture adjacent to the dairy cow farm. Half of the goat pasture was a wooded area, which was in continuity of a large mixed deciduous and coniferous forest dominated by beech trees. Goats that were found TBEV-antibody positive in the suspected and surrounding farms were aged between 2 and 10 years old, while cows sampled in the suspected and surrounding farms were aged between 1 and 10 years old.

When the epidemiological investigation led to determine that this farm was strongly suspected to be the source of the human contamination, all goats and dairy cows were confined into the stall, and cows were treated against ticks on May 26, 2020. Acaricide treatment of the flock buildings was also carried out. Serum and blood samples were collected as follows (Figure 1). On May 27, serum samples were collected from 30 goats. On June 2, 8, and 19, serum and blood samples were collected from all 56 goats from the farms. Serum samples were collected on the three dairy cows on June 2 and on the four suckling cows between July and August 2020. In addition, tank milk samples from goats and dairy cows were collected every week from June 2 to November 3. Individual milk samples were collected from the 55 lactating goats on June 8 and 19. In March 2021, a follow-up serological study was carried out on the entire goat herd among which 35 individuals were identical to 2020. Milk and sera were stored at 4°C, and blood samples were stored at −20°C until use.

#### From Animals in Surrounding Farms

To characterise the geographic distribution of the TBEV infections among the surrounding farms, one hundred and forty-two animals from five farms – two goat farms, one dairy cow farm, and two suckling cow farms – from which the animals have grazed in pastures located less than 2 km away from the infected farm’s pasture were investigated for the presence of TBEV antibodies. Serum samples of all animals available at the time of sampling were collected between June and August 2020.

#### Collect of Questing Ticks

Questing ticks were counted and collected from 10 a.m. to 6 p.m. on June 16, 2020 by dragging a 1-m^2^ white blanket. The pasture was divided into five geographic areas in which a total of 29 sub-areas were defined according to the habitat types: wooded areas (three sub-areas), wooded edge of the meadow (meadow-forest or meadow-wood ecotones, five sub-areas), hedgerows along the edge of the pasture or within the pasture (seven sub-areas), a meadow between 3 and 10 m away from the forest edge (four sub-areas) and from the hedgerows (four sub-areas), and a meadow more than 10 m from the edge (six sub-areas). The adjacent forest was divided into three sub-areas. The sampling scheme differed according to the sub-areas, with more effort made in wooded areas to collect more ticks for TBEV analysis. A total of twenty-eight 10-m-long transects were carried-out in either forest (18 transects) and wood (10 transects) sub-areas. In addition, the edges of the meadow and hedgerows were continuously checked for tick presence, and individuals were collected every 20-m-long transects. Within the meadow, five 20-m-long transects were realised. Because the dragging method is more appropriate for estimating the density of questing nymphs than those of larvae and adults, only the density of questing nymph per sub-area was estimated by evaluating the number of ticks collected per 100 m^2^ and its mean per habitat type was calculated.

We calculated that, for an estimated virus prevalence in nymphs of 0.2%, it would be necessary to collect and test for TBEV presence in a minimum of 1,000 nymphs to have a 95% chance to detect the virus (Cannon, 2001). Given that previous estimates of TBEV prevalence in the French endemic region (Alsace) ranged from 0.03 to 0.3% in nymphs and from 0.6 to 0.8% in adults (Perez-Eid et al., 1992; Bestehorn et al., 2018; Bournez et al., 2020), we conducted an additional collect of ticks in the forest to reach that critical minimum number. Back in the laboratory, ticks were identified to the genus level based on their morphology (Pérez-Eid, 2007). All questing nymphs and adults were washed in 70% ethanol, rinsed twice in distilled water, dried, and stored at −80°C until use for TBEV detection.

#### Small Mammal Trapping and Sampling of Feeding Ticks

For three consecutive nights from June 15 to 18, 2020, two hundred small mammal live-traps (14 × 14 Ugglan special no. 3, Grahnab, Sweden) were set up every 10 m along the pasture and within the wooded area of the pasture. Traps were baited with carrots and sunflower seeds. Caught animals were euthanised and identified at the genus level. The spleen and brain of small mammals were collected and put in nitrogen containers until storage at −80°C in the laboratory.

Ticks feeding on trapped animals were removed and identified to the genus level based on their morphology (Pérez-Eid, 2007). Potential undetected ticks were collected by removing the skin of the animals and storing them in individual bags for 48 h to let the ticks detach.

### Laboratory Analyses

#### Serological Tests

Antibodies against flaviviruses were detected using a multi-species commercial competitive enzyme-linked immunosorbent assay (cELISA; ID Screen^®^ West Nile Competition, ID Vet, Montpellier, France) based on purified whole West Nile Virus (WNV) antigen for the detection of antibodies directed against the envelope E protein common to all flaviviruses. Although this ELISA test is designed for WNV detection, it can be used for the detection of TBEV antibodies because of the high cross-reactivity among flaviviruses (Rushton et al., 2013; Beck et al., 2015, 2016; Bournez et al., 2019; Reusken et al., 2019). The test was performed according to the manufacturer’s instructions. The interpretation was modified for sera close to the doubtful and negative thresholds, with an extension of the doubtful interval, to ascertain the detection of sera with low-TBEV antibody levels by ELISA and then increase sensitivity. The sample was considered positive if <40%, doubtful between 40 and 72%, and negative if >72%.

In-house IgM-capture enzyme immunoassay (MAC-ELISA) with whole inactivated TBEV was performed as previously described (Peyrefitte et al., 2005) on all 30 goat sera sampled on May 27, 2020. MAC-ELISA was performed using rabbit anti-goat IgM antibodies (Bethyl Laboratories, Inc., Montgomery, United States).

Samples with positive and doubtful results in ELISA were then tested for the presence of specific neutralising antibodies against TBEV by micro virus neutralisation tests (MNTs, strain Hypr, Genbank ID U39292.1) as described in Beck et al. (2015). A serum sample was considered positive for TBEV if cells were protected at least at the serum dilution of 1:20.

#### RNA Extraction and Real-Time Tick-Borne Encephalitis Virus QRT-PCR

##### QRT-PCR Tests on Fresh Cheese

Cheese suspected to be infected with TBEV were stored at −80°C until analysis. The NRC for Arboviruses carried out TBEV genome screening following the protocol below. Dissolution of nearly 5 g of fresh or drier cheese was performed in phosphate buffer saline (PBS). Homogenisation steps were achieved to complete the process. RNA extraction was carried out using the QIAmp Viral RNA Mini Kit (Qiagen, Paris, France) according to manufacturer’s instructions. Five microlitres of the eluted RNA were used to execute a quantitative RT-PCR with primers and probe targetting TBEV genome (sequences available on request) using the SuperScript™ III Platinum™ One-Step qRT-PCR amplification kit (Thermofischer, Paris, France) on LightCycler^®^ 2.0 Instrument (Roche Life Science, Mannheim, Baden-Wurttemberg, Germany).

Confirmation of TBEV-infected cheese was conducted at the food-safety national reference laboratory, ANSES, Maisons-Alfort. The method used to recover TBEV from fresh cheese was based on the use of proteinase K as previously described in Hennechart-Collette et al. (2017). After enzymatic digestion, total nucleic acid extraction was carried out using the NucliSENS^®^ easyMAG™ platform according to the manufacturer’s instructions (bioMérieux). The primers and probe used to detect TBEV by RT-qPCR have been already described in the literature by Gondard et al. (2018).

##### QRT-PCR Tests on Goats, Cows, and Small Mammal Samples

Goat and dairy cow tank milks, goat individual milk samples, goat or cows ethylenediaminetetraacetic acid (EDTA) blood, and the spleen and brain of small mammals suspected to be infected with TBEV were stored at 4°C (milk), −20°C (EDTA blood), or −80°C (organs) until analysis. RNA extraction was performed using the MagVet™ Universal Isolation kit (Lifetechnologies, Saint-Aubin, France) on the King Fisher automat (ThermoFisher Scientific, Paris, France). Five microlitres of each RNA extract were subjected to qRT-PCR with primers and probe targetting the NS5 gene described in Gondard et al. (2018) using the AgPath-ID™One-Step RT-PCR Reagents kit (Lifetechnologies, Saint-Aubin, France) and cycling conditions as follows: reverse transcription for 10 min at 45°C; denaturation of cDNA 10 min at 95°C and 45 cycles of 15 s at 95°C, and 1 min at 60°C. The detection limit of the qRT-PCR, which is the lowest number of copy genome detected for a known dilution in 95% of cases, performed on goat milk samples was estimated at 1.10e4 TCID50/ml corresponding to a Ct value of 34.

##### QRT-PCR Tests on Questing Ticks and Descriptive Analysis of Tick-Borne Encephalitis Virus Infection in Ticks

Questing ticks were analysed to detect TBEV RNA. Adult ticks were individually analysed and nymphal ticks were analysed in pools of 30 ticks maximum per sub-area. RNA was extracted using the Nucleospin RNA II extract kit (Macherey-Nagel, Düren, Germany) as described in Bournez et al. (2020) and were screened for TBEV by qRT-PCR targetting the NS5 as described in Gondard et al. (2018). Because TBEV infection prevalence in ticks is lower than 1% in endemic regions of France, the prevalence in nymphs was expressed as the minimum infection rate per 100 tested (MIR) based on the assumption that a single tick was positive within a positive pool. We calculated the overall MIR of TBEV in nymphs (the number of positive pools divided by the overall number of nymphs tested × 100) and TBEV prevalence infection in adults (the number of positive adult ticks by the overall number of adult ticks tested ×100). Their 95% CIs were calculated following a binomial distribution. We estimated the density of infected nymphs per habitat by multiplying the% MIR by the mean density of questing nymphs per habitat.

##### Virus Isolation From Contaminated Milk and Infected Ticks

Contaminated milk (500 μl) or tick grindings (100 μl/homogenates) positive for TBEV were diluted in serum-free Dulbeccos’ Modified Eagle Medium (DMEM) culture, inoculated in a T25 flask seeded with Vero NK cells (ATCC: CCL81™) 24 h earlier, and washed with DMEM before inoculation. Following an incubation time of 1 h 30 at 37°C with 5% CO_2_, cells were washed twice with PBS, and a complete medium (DMEM + 1% penicillin- streptomycin + 1% sodium pyruvate + 5% fetal calf serum) was added. The cells were observed every day from day 3 to day 7 post-infection (pi). As soon as cytopathic effects (CPE) were detected, the supernatant was collected, clarified, and stored at -80°C. RNA extraction was performed using the MagVet™ Universal Isolation kit (Lifetechnologies, Saint-Aubin, France) on the King Fisher automat (ThermoFisher Scientific, Paris, France). RNA extracts were subjected to TBEV RT-qPCR as described above to confirm TBEV detection.

##### Full-Length Tick-Borne Encephalitis Virus Genome Assembly Performed From Contaminated Milk and Infected Ticks

Full-length virus genome sequencing was conducted on cDNA obtained from contaminated milk and infected ticks. Multiplex primers were designed using a Primal scheme^1^ with default parameters. A multifasta file with reference genomes NC_001672.1, KF151173.1, MG589938.1, KC835596.1, MK801808.1, MK801803.1, MG210948.1, KX966399.1, and KP716974.1 was used to generate primers. Their sequences are listed in Table 1 (Supplementary Data) (scheme.primer.tsv). A multiplex PCR method for targetted enrichment was adapted for library preparation and MinION sequencing of TBEV genome as previously described for Zika (WHO Control Reference 11474/16) and Chikungunya viruses (PEI N11602) in Quick et al. (2017).

**TABLE 1:** Summary of the test results carried out on serum and milk samples collected from the goats of the suspected farm.

Date	Serum tested by serological tests			Individual milk tested by qRT-PCR			Tank milk tested by RT-PCR	
	No.	No. TBEV positive	% TBEV-positive	No	No. TBEV positive	% TBEV-positive	Yes/no	Ct value
27/05	30	6	20	0			No	
02/06	56	11	20	0			Yes	35
09/06	56	13*	23	55	3	5.5	Yes	Neg
23/06	56	13*	23	55	0	0	Yes	Neg
07/07	0			0			Yes	Neg
15/07	0			0			Yes	Neg
28/07	0			0			Yes	Neg
04/08	0			0			Yes	Neg
11/08-03/11	0			0			Yes	Neg
2021	53	8	15	0			No	

*Serum samples were initially tested by a competitive ELISA. Then, positive and doubtful ELISA samples were tested by specific-TBEV virus micro-neutralisation tests (MNT). A serum sample was considered TBEV-seropositive when MNT was positive.*

*Two goats were MNT negative at one of these two dates but considered TBEV positive as these were clearly positive by MNT on 2020/06/02. Moreover, both of them excreted TBEV in the milk.

Sample barcoding was performed with 14 barcodes (NB01 to 14) with the native barcoding genomic DNA protocol using the ligation sequencing kit SQK-LSK109 (Oxford Nanopore Technologies, Oxford, United Kingdom) and the Native barcoding expansion kits EXP-NBD104 and EXP-NBD114 (Oxford Nanopore Technologies, Oxford, United Kingdom). Two barcodes were assigned per sample depending on the pool of primers used (either pool 1 or 2) (Supplementary Table 2). A Flongle flow cell FLO-FLG001 was used for sequencing (Oxford Nanopore Technologies, Oxford, United Kingdom).

FAST5 reads were base called offline using guppy_basecaller v5.0.7. from Oxford Nanopore.^2^ Subsequent FASTQ files were demultiplexed using guppy_barcoder using the –trim-barcodes flag. Twenty-five nucleotides were trimmed at both 5′ and 3′ ends in resulting FASTQ files to remove primers with a maximum length of 700 bp using NanoFilt v2.6. The sequences obtained with the two barcodes corresponding to a unique sample were concatenated into one FASTQ file. Each file was mapped to the reference NC_001672.1 using minimap2 v2.20 (Li, 2018). Consensus FASTA files were generated after four error correction steps, including three polishing steps of racon v1.4.20^3^ and one polishing step with medaka v1.4.3.^4^ Trimmed reads were mapped back to the assembly using minimap2, and coverage statistics were calculated on the sorted BAM file using SAMtools v1.12 (Li and Durbin, 2009). Multiple alignments of the nucleotide sequences were performed using the ClustalW algorithm to extract a high-quality full-length genome sequence.

##### Full-Length Tick-Borne Encephalitis Virus Genome Assembly Performed From Contaminated Cheese

Full-length virus genome sequencing was conducted on cDNA obtained from contaminated cheese using the ProtoScript^®^ II Reverse Transcriptase kit (New England Biolabs, MA, United States) with 50 μM random hexamers (Invitrogen) (7:1 ratio) for 5 min at 65°C (Bournez et al., 2020). The full-length genome was amplified (multiplex PCR) with Q5^®^ High-Fidelity 2X Master Mix kit (New England Biolabs, MA, United States) and a set of primers designed using the “Primal Scheme” program (see text footnote 1; Quick et al., 2017). The list of primers used for sequencing is presented in Supplementary Table 3. Sequencing analysis was performed using the S5 Ion torrent technology v5.12 (ThermoFisher Scientific, Paris, France) as previously described (Driouich et al., 2019). The consensus sequence was obtained after a trimming step of reads (reads with quality score < 0.99, and length < 100 bp were removed, and the thirty first and thirty last nucleotides were removed from the reads). Mapping of reads to a reference (determined following blast of *De Novo* contigs) was realised using CLC genomics workbench software v.20 (Qiagen, Hilden, Germany). A *de novo* contig was also generated to ensure that the consensus sequence was not impaired by the reference sequence. For genomic regions for which no sequence was obtained using this approach, cDNA was also generated and amplified using™ the SuperScript IV One-Step RT-PCR System kit following the supplier’s recommendations (Invitrogen). PCR mixes (final volume 50 μl) contained 3 μl of cDNA, 2.5 μl of each primer (final concentration of 0.5 μM), and 25 μl of 2X Platinum™ SuperFi™ RT-PCR Master Mix. Amplification was performed with the following conditions: 50 s at 50°C, 2 min at 98°C, then 40 cycles of amplification (10 s at 98°C, 10 s at 66°C, and 30 s at 72°C), and a final extension of 5 min at 72°C. PCR products were verified by gel electrophoresis and pooled. Sequencing analysis was performed as described above. The consensus sequence was obtained using the reads from both runs as described above.

##### Phylogenetic Analyses

The novel reference genome sequence was aligned against the known TBEV viral diversity. Nucleotide and protein percentage of identity were calculated with Clustal Omega (Madeira et al., 2019). Phylogenetic analyses were conducted on the full-length open reading frame. Phylogenetic trees were then inferred using the maximum likelihood method implemented in PhyML (version 3.0) (Lefort et al., 2017) using the best-fit model and best of nearest neighbor interchanges (NNI) and Subtree Pruning and Regrafting (SPR) branch swapping. Support for nodes on the trees was assessed using an approximate likelihood ratio test (aLRT) with the Shimodaira-Hasegawa-like procedure. Trees generated using the Neighbour–Joining and Maximum Likelihood methods implemented in MEGAX gave identical results.

## Results

### Tick-Borne Encephalitis Virus Detection in Fresh Cheese Produced in the Suspected Farm

On June 2, the NRC Arbovirus detected one cheese that is tested positive for TBEV among seventeen. This cheese, produced on April 28, was collected on May 26 in a patient home during the investigation time. The local authorities suspected a food-born source of contamination as patients developed meningoencephalitis after consuming goat cheese produced on the same farm. The presence of TBEV genome was confirmed by the ANSES food-safety laboratory (Maisons-Alfort). The food-borne origin of TBEV contamination was therefore confirmed. On July 10, seventy-four cheese given by the producer were analysed and virus genome was detected in six of them.

### Tick-Borne Encephalitis Virus Infection and Exposure in Suspected Farm Livestock

**FIGURE 2:**
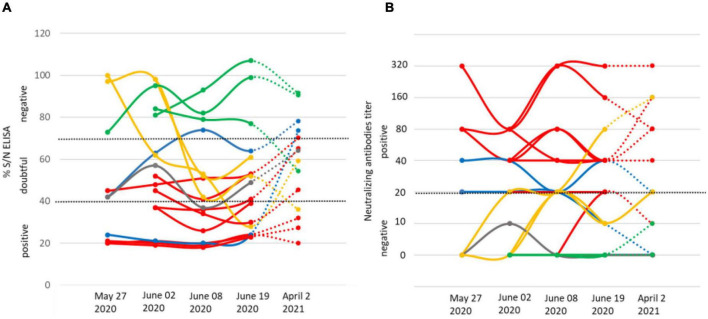
IgG levels **(A)** and micro-neutralisation antibody levels **(B)** in goats on four dates in 2020 (May 27, June 2, June 8, and June 19) and on April 2, 2021. The threshold levels applied are indicated as dashed lines. We provide examples of seronegative individuals (green) and show results for the individual that returned seropositive *via* ELISA competition, but returned negative in neutralising antibody titre (grey), the three individuals that seroconverted after May 27 (yellow), the eight seropositive individuals (red), and the two individuals that returned negative IgG competition results in 2021 (blue), one of which maintains neutralising antibodies.

IgG and IgM antibodies against TBEV were detected using the ELISA tests (Igs competition and IgM capture) and MNT. On May 27, six goats out of thirty were TBEV-seropositive as assessed with competition ELISA and MNT. One of them had anti-TBEV IgM antibodies. The following weeks, after their placement in a stable to prevent further tick bites, all 56 goats were tested for a complete assessment of the TBEV exposure rate. Eleven goats were TBEV-seropositive on June 2 (20%) (with one seroconversion in the 30 goats collected on May 27) and 13 (23%) on June 9 (with two more seroconversions) (Figure 2). Goat seroconversions were confirmed by MNT (Figure 2, Table 1, and Supplementary Data). Overall, the ELISA results were confirmed by MNT with the exception of a single individual that appeared positive based on ELISA competition but did not develop neutralising antibodies (Figure 2). The seroconversion of three goats between May 27 and June 9 indicates that infection occurred very recently. Yet, none of the animals had high viremia given that all qRT-PCRs conducted on blood samples were negative (collected on June 2, 9, and 19). ELISA competition conducted in 2021, about 1 year after the initial infection, showed a decrease in IgG levels for most seropositive individuals (10/13), but the level of neutralising antibodies remained high. Two individuals that were TBEV-seropositive in 2020 turned negative based on competition ELISA in 2021, and one of those had no remaining neutralising antibodies according to MNT (Figure 2). Within the same farm, one of the three dairy cows and two of the four suckling cows which had grazed in the same or in the adjacent pasture as the goats were TBEV seropositive. No virus genome was detected in the blood of these animals.

### Tick-Borne Encephalitis Virus Detection in Goat Milk at the Suspected Farm

The presence of the TBEV genome was detected in the tank of goat milk collected on June 2, but it was absent in the tank of cow milk (Table 1). The virus was not detected in either tank thereafter (between June 8 and November 3). The presence of TBEV was tested in the milk of individual goats collected on June 8 and 19. TBEV genome was detected in the milk of three goats on June 8 (23% of the thirteen seropositive animals), indicating that they continued excreting viruses 14 days after their confinement. Meanwhile, two individuals were TBEV-seropositive on May 27 and one seroconverted between June 2 and 8. On June 19, all individual milk samples returned negative results (Table 1).

### Seroprevalence Survey in Surrounding Farms

**FIGURE 3:**
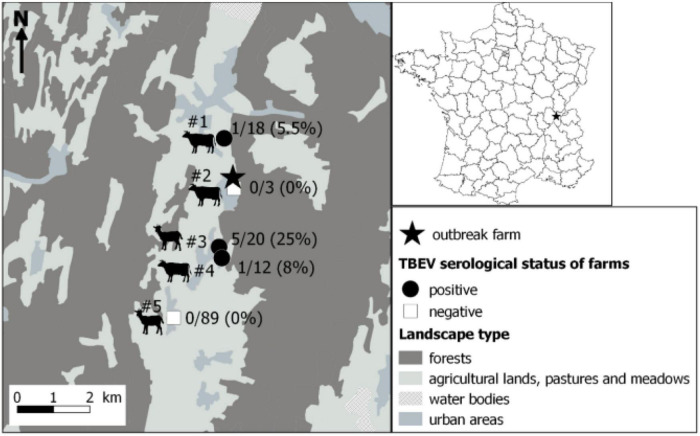
Localisation of the farm responsible for the tick-borne encephalitis cluster through alimentary route (star) and results of the serological survey conducted in surrounding farms. Serum samples were first tested by a competitive ELISA. Then, positive and doubtful ELISA samples were tested by specific tick-borne encephalitis virus (TBEV) micro-neutralisation tests (MNT). A serum sample was considered TBEV-seropositive when MNT was positive. Dairy cows, suckling cows, goats, suckling cows, and goats were found in farms #1 to #5, respectively.

Within a couple of kilometers away from the suspected farm, five farms that were hosting cows and goats were identified, and sera from all individuals were collected and processed as above. Few seropositive animals were found in three of the five farms, with one to five seropositive animals and a TBEV seroprevalence ranging from 5.5 to 25% (Figure 2 and Table 2). Animals from the suspected farm and from farms #1 and #3 within which seropositive animals were found have all grazed in meadows located in close proximity to the same forest (Figure 3). On farm #4, a single 10-year-old cow was TBEV-seropositive, but it was not possible to trace back where the cow had grazed in the past.

**TABLE 2:** Number of questing ticks collected and mean density per habitat.

Habitat	No. sub-areas	Sampled surface (m^2^)	No. nymphs	No. female	No. male	Density of nymphs (/100 m^2^)	
						mean	Range
Forest	3	540	382	10	20	71.5	64.2–81.3
*Additional collection*	/	/	*285*	*7*	*5*	/	/
Pasture							
Wooded area	3	300	105	6	3	31.7	20.0–50.0
Wooded edge	5	400	94	2	2	24.0	5–56.7
Hedgerows	7	870	4	0	0	0.3	0–1.1
Meadow between 3 and 10 m from the wooded edge	4	530	35	2	1	4.5	0–8.8
Meadow between 3 and 10 m from the hedgerow	4	400	2	0	0	0.3	0–1.1
Meadow > 10 m from the edge	6	600	0	0	0	0	0

### Tick-Borne Encephalitis Virus Presence in Ticks and Rodents Within and Around the Pasture

The goats have grazed in only one pasture adjacent to the dairy cow farm. Half of the goat pasture was a wooded area which was in continuity of a large mixed deciduous and coniferous forest dominated by beech trees.

#### Detection of Tick-Borne Encephalitis Virus in Ticks

A total of 120 larvae, 907 nymphs, 27 females, and 31 males of questing *Ixodes* spp. and 1 female of *Dermacentor reticulatus* were collected within the pasture and in the adjacent forest. Within the pasture, ticks were only found in the wooded area, particularly along the wooded edge and hedgerow and in the meadow situated less than 10 m away from the forest edge. The density of questing *Ixodes* spp. nymphs was the highest in the forest with a mean of 71.5 nymphs/100 m^2^, then in the wooded areas, in the wooded edge of the pasture with a mean of 24–32 nymphs/100 m^2^, and, finally, in the meadow with a mean of 4.5 nymphs/100 m^2^ (Table 2).

Two pool of nymphs - from the forest and from the meadow located between the wooded area of the pasture and the forest - and one male from the forest were subsequently tested positive for TBEV RNA. Overall, the MIR was 0.22% (IC_95%_: 0.03–0.80%) in nymphs, and the infection rate was 1.8% (IC_95%_: 0.1–9.9%) in adults. The estimated density of infected nymphs was the highest in the forest with 0.15 infected nymphs/100 m^2^, then in the wooded area and in the wooded edge of the pasture with 0.05–0.06 infected nymphs/100 m^2^.

#### Absence of Tick-Borne Encephalitis Virus in Captured Small Mammals

In addition, small mammals were caught around the pasture to assess TBEV presence. In total, ten small mammals were captured: nine shrews (*Sorex* spp.) and one bank vole (*Myodes glareolus*). Only the bank vole was captured at the edge of the forest. Larvae of *Ixodes ricinus* were, respectively, observed on the bank vole (five larvae) and on two shrews (two larvae). All animal organs (spleen and brain) were negative to TBEV as assessed by qRT-PCR.

### Phylogenetic Analysis

**FIGURE 4:**
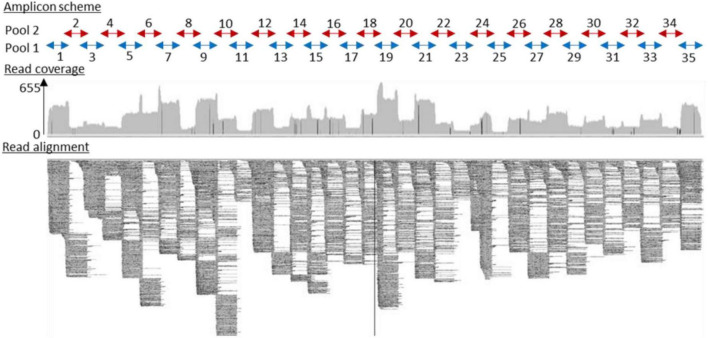
Full-length TBEV genome sequence (GenBank accession number: OL441148) was obtained from sequencing 35 overlapping amplicons in two multiplex reactions. Read coverage varied along the sequence length, but a full-length genome sequence was successfully recovered.

Three RNA samples were extracted from two TBEV-positive pools of nymphs and one TBEV-positive male tick, and RNA samples obtained from three individual infected-goat milk were subjected to sequencing. Our amplicon-based sequencing protocol allowed us to successfully sequence the TBEV genome from RNA extracted from milk and ticks (Figure 4). The full-length genome sequence obtained from milk and ticks was similar to the full-length genome obtained from the contaminated cheese collected during the investigation led by a local authority agency. Sequences obtained from the milk sample, and the virus isolate obtained from ticks were 100% identical except for single nucleotide insertions and deletions that are most likely due to sequencing errors.

**FIGURE 5:**
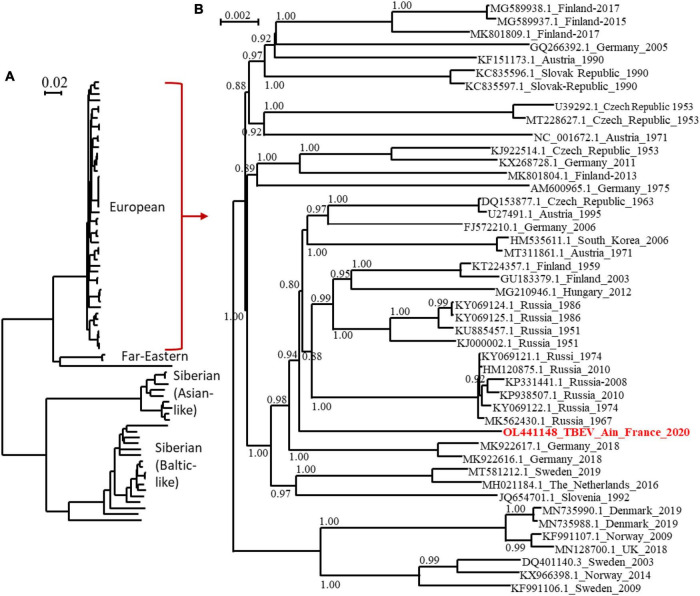
Phylogenetic tree of TBEV complete open reading frames that were available in GenBank and the TBEV-Ain-France-2020 obtained in the present study (marked red) using all sequences **(A)** or those corresponding to the European subtype only **(B)**. The two trees were inferred in PhyML using the Le Gascuel (LG) substitution model. Branch points indicate that results of Shimodaira-Hasgawa branch test > 0.8. Scale bar shows the number of nucleotide changes. Virus isolate names are given as follows: accession number from NCBI_ country to name_year.

The full-length genome of the new viral strain was sequenced and compared to previously characterised TBEV genome sequences. The TBEV_Ain_France_2020 had limited homology (98.4% identity) to the partial NS5 of the TBEV from the Alsace region (AF0910) (Supplementary Data). The nucleotide sequence presents a 98.8% identity to the same partial NS5 and between 98 and 98.07% identity to the full-length genomes of TBEV isolates detected in Russia, Germany, Austria, Czech Republic, and the Netherlands with the most ancient isolate dating from 1951 in Russia. The polyprotein sequence presented up to 99.30 and 99.24% identity to MK922616 and MK922617 isolated from ticks collected in the German federal state of Lower Saxony in 2018. Our phylogenetic analyses confirmed that TBEV_Ain_2020 (OL441148) belongs to the European subtype (TBEV-Eu3) and is most closely related to TBEV strains recently isolated in bordering countries and Eastern Europe (Figure 5).

## Discussion

Food-borne TBE outbreaks had been reported in several endemic countries in Central Europe at a regular frequency, particularly in countries where the consumption of traditional raw milk products is popular (Kerlik et al., 2018). France is on the border of the TBEV geographic range, and cases resulting from tick bites are reported very occasionally. The first occurrence of TBE-food-borne outbreak in France in 2020 has led to an unexpected high number of cases. Forty-three patients have developed clinical symptoms compatible with TBE, and all but one had consumed raw goat milk and/or cheese products originating from a single producer, while 41 cases could be confirmed by detection of TBEV-specific antibodies in sera and/or in cerebrospinal fluid. A health alert was triggered within a month after the first patient showed symptoms, and an in-depth investigation of TBEV presence was conducted in the farm suspected to be the source of contamination and in animals in surrounding farms. We applied a “one health” approach, combining environmental, veterinary, virology, and food microbiology approaches to identify the origin of infection and provide a range of epidemiological information of significance to public health decision-makers. We characterised the TBEV strain circulating in Ain, detected viral genome presence in a batch of cheese, goat milk, and in questing ticks, demonstrated recent infection of goats, assessed the enzootic hazard for TBEV exposure, and identified a wooded TBEV-focus area. This integrative investigation provides information that would allow the farmer and authorities to assess the risk of infection and develop control measures.

The initial investigation of seroprevalence (23%) in goat flocks at the suspected farm reveals that the animals had been moderately exposed to the virus these past few years. In neighboring areas where TBEV is endemic, such as Switzerland and Germany, seroprevalence varies from 1 to 80% with median range value of 10–20% (calculated from Klaus et al., 2012; Klaus et al., 2014; Rieille et al., 2017; Casati Pagani et al., 2019). Seroconversion results, presence of IgM antibodies, and detection of the virus genome in milk indicate that infection was very recent for half of the animals that had been exposed to the virus (6 animals over 13). IgM was detected in one individual. Three individuals seroconverted after entering the stable, indicating that TBEV infection occurred a few days before they entered the stable. Indeed, seroconversion in goats and sheep occurs in 6–10 days (Van Tongeren, 1955; Klaus et al., 2014; Paulsen et al., 2019). One of these individuals and two others excreted viruses in milk 14 days after entering the stable. These results also support a recent infection given that virus excretion in goat and sheep milk is detected from 2 to 23 days after infection (Gresikova, 1958; Gresíková et al., 1975; Balogh et al., 2010, 2012). So far, information is relatively scarce on the infection dynamics in ruminants, especially in naturally infected animals. Our investigation confirmed a long-lasting excretion of the virus in milk in naturally infected goats since three individuals excreted the virus into milk for over 14 days post-infection. The date of the seroconversion of the additional seven TBEV-seropositive goats and two cows from the same farm is unclear. Previous studies suggest that TBEV-antibodies may persist up to 6 years, but the level of IgG produced decreases over time (Klaus et al., 2014, 2019). In agreement, even though the level of neutralising antibodies remain high a year later, we observed a decrease in the level of IgG detected by ELISA. Thus, the high level of IgG, as measured by ELISA competition, indicates that the remaining animals have been likely infected or re-infected by the virus in 2020. Since no virus excretion has been observed in five experimentally pre-immunised goats (Balogh et al., 2012), only naïve individuals would have excreted TBEV in milk. The high recent infection rate among naïve individuals of the flock would explain the high virus load detected in tank milk in June. Milk and cheese viral load were determined using a genome quantification assay, whereby milk sample concentration was estimated based on cycle threshold (Ct) values derived from measurements of serial dilutions of infectious TBE viral particles with known concentrations (from 10^8^ to 10^3^ TCID50/ml). We evaluated a minimal detection of infectious viral particles at 10^4^ TCID50/ml of milk and 10^5^ TCID50/ml after dissolution of 2.5 g of cheese. In ticks, the mean viral copy number lies between 2 × 10^2^ and 4.8 × 10^3^ RNA copies per sample (Liebig et al., 2020, 2021). Our data confirmed the high virus load detected in tank milk. Moreover, the virus was detected in seven cheese (over 91 tested). Among them, one contaminated cheese was produced on April 28. This is outstanding, especially when we consider that cheese was kept for several days at 1–5°C by consumers or producers before being analysed, which may have reduced the persistence of TBEV load and hence its detection.

The high recent infection rate of the goat flock suggests regular contact between TBE-infected ticks and goats in spring 2020, when ticks started to be active. We found infected questing nymphs and adults in the wooded edge of the pasture (in the meadow between the pasture wood and the forest) and in the forest nearby. With an MIR of 0.22% (IC_95%_: 0.03–0.80%) in nymphs and an infection rate of 1.8% (IC_95%_: 0.1–9.9%) in adults, it appears that TBEV is well established in the forest near the pasture. Indeed, while the densities and prevalence of infected questing ticks may not be deemed exceptionally high compared to what is seen in endemic countries (Süss, 2011), it is similar to the highest values observed in an endemic foci in Alsace region, East France (Bournez et al., 2020). Since we captured a few adult ticks (probably because the dragging method is not well suited to capture this stage), the density of infected adults was estimated with a low precision and hence might be higher than those observed in Alsace. We did not detect TBEV RNA in small mammals trapped in the pasture, but we probably missed infected individuals since we only trapped a few rodents. Indeed, the year 2020 was probably a low phase of the usual yearly fluctuating rodent population. Overall, the results suggest that the presence of a large wooded area within the pasture frequently used by goats as a shelter as well as of a long pasture-forest edge where tick densities are relatively high have likely favoured the contacts between goats and TBEV-infected ticks. The forest may also represent a source of frequent introduction of infected ticks into the pasture through the movements of tick-feeding hosts.

This cluster was surprising by its location. It occurred in the Ain department in France where TBEV human cases had never been reported. Our investigation in surrounding farms also revealed that TBEV was also present in various pastures surrounding the same single forest where we found infected ticks over at least 2 km. In three of these farms, seroprevalence varied between 5.5 and 25%. With the exception of one individual (for which it was not possible to track back the pasture in which it had grazed), all seropositive animals had likely grazed within a pasture adjacent to the same forest. These results indicate that the virus is well established in the area and, probably, in a larger extent in the Ain department. Because of the absence of an active TBEV surveillance system assessing its distribution in France and potential misleading of TBE diagnosis, it is impossible to conclude whether the presence of TBEV in the department is ancient or whether it was recently introduced. In addition, diagnosis of human TBE cases was initially delayed as TBE serology was not systematically researched among patients presenting with meningitis or meningoencephalitis. Similarly, the presence of TBEV in centre France, in Loire, and Haute-Loire departments detected for the first time in 2017 and 2018 (Herpe et al., 2007; Velay et al., 2018) is impossible to date. Given that the life cycle of ticks involves generally one meal per stage per year, we can presume that TBEV-infected ticks have been present in the environment for at least 1 or 2 years before the occurrence of human cases. Evidence suggest that the geographic range of TBEV is currently expanding in Europe, especially in Western and Northern Europe (Csángó et al., 2004; Donoso Mantke et al., 2011; Rieille et al., 2017; Andersen et al., 2019; Casati Pagani et al., 2019; Smura et al., 2019; Alfano et al., 2020; Ullrich et al., 2021). This expansion also have occurred in France. For instance, in a recent expansion of TBEV in higher altitudes, other previously unaffected areas or ancient affected areas have been observed in several France’s neighbouring endemic countries, including Germany (Dobler et al., 2019; Hellenbrand et al., 2019), Switzerland (Rieille et al., 2017; Casati Pagani et al., 2019; Desgrandchamps et al., 2019), and Italy (Alfano et al., 2020). The virus was also detected for the first time in the United Kingdom in 2018 (Holding et al., 2020) and in the Netherlands in 2015 (Jahfari et al., 2017). TBEV expansion is believed to be driven by climate, landscape, and anthropogenic changes (Randolph, 2010; Jaenson et al., 2012; Holding et al., 2020). Moreover, the first occurrence of TBEV in new areas in France since 2017 might be the results of exceptional high densities of infected ticks these past 5 years in endemic areas, which may have led to higher dispersal rates of infected ticks into new areas. Indeed, although TBE human incidence usually exhibits significant oscillations over time, in 2016–2020, it was persistently high in numerous endemic countries of Western and Central Europe (Germany, Switzerland, Czech Republic, Slovakia, and Italy), including France (Dobler et al., 2019). This may partly be caused by high densities of infected ticks during those years. A high density of infected ticks during several years in endemic areas may increase the frequency of medium or long-distance movements of infected ticks on their host (birds, cervids) and led to a more successful introduction of the virus into new areas. Alternatively, it is also possible that TBEV has been circulating in Ain department undetected for decades, and the 2020 outbreak could be the result of suitable conditions for its detection. Indeed, TBE cases are likely underdiagnosed in France given that human clinical cases are rare and local clinicians are unfamiliar with the disease (infection often asymptomatic, not yet notifiable disease in April 2020, low virus circulation, few TBEV foci, few human visits in infected areas) (Dollat et al., 2021). The year of 2020 was exceptional with a very high human incidence observed in numerous endemic European countries. In Switzerland and Germany, the notable increase of TBE cases observed in spring 2020 was partly associated with higher recreational activities in risky endemic areas around people’s homes during the COVID-19 containment measures (Steffen et al., 2020; Ullrich et al., 2021). In France, the COVID-19 containment measures may have also indirectly increased the consumption of local products. Hence, the emergence of a cluster of food-borne TBE cases in a restricted geographical area that was easier to detect. However, this was not the sole factor as an upsurge of infected tick densities in 2020 likely contributed to the rise of human incidence. Indeed, models forecasting the human TBE incidence based on demographic parameters, large-scale atmospheric circulation patterns, the fructification of the European beech (*Fagus sylvatica*) 2 years prior, and the national TBE vaccination coverage already predicted an increase in TBE incidence in 2020 in Germany, Switzerland, and Austria (Rubel and Brugger, 2020, 2021). In Germany, an unusually high density of infected adult ticks has also been observed in 2020 in some places (PROMED, 2020).

We provide the first TBEV isolate responsible for a source of dietary contamination in France, which will facilitate experimental studies. The genome sequences obtained were used to conduct phylogenetic analyses using the complete open reading frame. We revealed that the TBEV_Ain_France_2020 belonged to the European TBEV subtype, but do not cluster with the strain circulating in the Alsace region and to other isolates from Europe, with the closest relatives being isolates from Germany, Austria, Czech Republic, The Netherlands, and Russia. In all actuality, we do not know enough about the distribution of TBEV in France and the genetic diversity of the TBEV genome in Europe. The distinct genome of the French isolate would tend to indicate that this isolate has not recently spread to France but has remained undetected and evolved locally for some time. Further studies in the department and neighbouring regions are needed and are being set up to further characterise the focus-specific genetic diversity of TBEV and to confirm or disprove the ancient origin and geographical extension of this isolate.

In conclusion, the cluster of TBE alimentary cases in Ain was unexpected. It surprised clinicians and local health authorities given the number of infected patients. The high recent infection rate of the goat flock probably led to a high virus load in tank milk in April and May and facilitated infection by consumption of raw cheese. In addition, the COVID-19 containment measures in spring 2020 may have also indirectly increased the consumption of local products and, hence, the risk of food-borne TBE cases. The Ain outbreak also highlighted the need for improving surveillance, detection, and prevention of TBE in France. Clearly, a better understanding of TBEV distribution in France, of local virus dynamic, and of food-borne contamination with better identification of raw milk products being at risk is needed to better adapt surveillance and prevention measures. This is all more important as France produces each year 40,000 tonnes of raw milk-products and cheese. In addition, the local production and consumption of traditional delicacies is increasing. TBEV is now a notifiable disease in France, facilitating the systematic report of human cases and sensitising medical communities for diagnosis of humans showing encephalitis in France. In endemic regions and those surrounding affected areas, patients with meningitis and encephalitis in the absence of another etiological diagnosis should be more systemically screened for TBEV infection (Herpe et al., 2007; Velay et al., 2018; Botelho-Nevers et al., 2019; Blanchon et al., 2020). Surveillance could also include serological testing for anti-TBEV antibodies in animal blood or in milk tanks to allow early detection of virus foci (Imhoff et al., 2015; Rieille et al., 2017; Wallenhammar et al., 2020; Bauer et al., 2021). Finally, in focus areas, TBE vaccination could provide protection from disease caused by infections from tick bites or consumption of virus-contaminated dairy products (Chernokhaeva et al., 2018; Kubinski et al., 2020; Chitimia-Dobler et al., 2021).

## Data Availability Statement

The datasets presented in this study can be found in online repositories. The names of the repository/repositories and accession number(s) can be found in the article/Supplementary Material.

## Ethics Statement

Ethical review and approval was not required for the animal study because the species captured during this study are neither protected in France nor included in the International Union for Conservation Nature Red List of threatened species and, therefore, no special authorisation was needed for trapping, according to French law. Animal trapping took place with permission from the landowners. All efforts were made to minimise animal suffering. The traps that we used are designed not to stress or harm the animals. Small mammals were euthanised by authorised experimenters according to French law and to the European guidelines (AVMA Guidelines for the euthanasia of Animals: 2020 editions). Written informed consent was obtained from the owners for the participation of their animals in this study.

## Author Contributions

All authors contributed to the article and approved the submitted version.

## Conflict of Interest

The authors declare that the research was conducted in the absence of any commercial or financial relationships that could be construed as a potential conflict of interest.

## Publisher’s Note

All claims expressed in this article are solely those of the authors and do not necessarily represent those of their affiliated organizations, or those of the publisher, the editors and the reviewers. Any product that may be evaluated in this article, or claim that may be made by its manufacturer, is not guaranteed or endorsed by the publisher.

## Footnotes

1
http://primal.zibraproject.org

2
https://nanoporetech.com

3
https://github.com/isovic/racon

4
https://github.com/nanoporetech/medaka

## References

[B1] Alfano N. Tagliapietra V. Rosso F. Ziegler U. Arnoldi D. Rizzoli A. (2020). Tick-borne encephalitis foci in northeast Italy revealed by combined virus detection in ticks, serosurvey on goats and human cases. *Emerg. Microbes Infect.* 9 474–484. 10.1080/22221751.2020.1730246 32100632 PMC7054962

[B2] Andersen N. S. Larsen S. L. Olesen C. R. Stiasny K. Kolmos H. J. Jensen P. M. (2019). Continued expansion of tick-borne pathogens: tick-borne encephalitis virus complex and *Anaplasma phagocytophilum* in Denmark. *Ticks Tick-borne Dis.* 10 115–123. 10.1016/j.ttbdis.2018.09.007 30245088

[B3] Balogh Z. Egyed L. Ferenczi E. Bán E. Szomor K. N. Takács M. (2012). Experimental infection of goats with tick-borne encephalitis virus and the possibilities to prevent virus transmission by raw goat milk. *Intervirology* 55 194–200. 10.1159/000324023 21325791

[B4] Balogh Z. Ferenczi E. Szeles K. Stefanoff P. Gut W. Szomor K. N. (2010). Tick-borne encephalitis outbreak in Hungary due to consumption of raw goat milk. *J. Virol. Methods* 163 481–485. 10.1016/j.jviromet.2009.10.003 19836419

[B5] Bauer B. U. Könenkamp L. Stöter M. Wolf A. Ganter M. Steffen I. (2021). Increasing awareness for tick-borne encephalitis virus using small ruminants as suitable sentinels: Preliminary observations. *One Health* 12:100227. 10.1016/j.onehlt.2021.100227 33732862 PMC7937955

[B6] Beck C. Desprès P. Paulous S. Vanhomwegen J. Lowenski S. Nowotny N. (2015). A high-performance multiplex immunoassay for serodiagnosis of flavivirus-associated neurological diseases in horses. *BioMed. Res. Int.* 2015:678084. 10.1155/2015/678084 26457301 PMC4589573

[B7] Beck Y. Fritz R. Orlinger K. Kiermayr S. Ilk R. Portsmouth D. (2016). Molecular basis of the divergent immunogenicity of two pediatric tick-borne encephalitis virus vaccines. *J. Virol.* 90 1964–1972. 10.1128/JVI.02985-15 26656681 PMC4734018

[B8] Bestehorn M. Weigold S. Kern W. V. Chitimia-Dobler L. Mackenstedt U. Dobler G. (2018). Phylogenetics of tick-borne encephalitis virus in endemic foci in the upper rhine region in France and Germany. *PLoS One* 13:e0204790. 10.1371/journal.pone.0204790 30335778 PMC6193627

[B9] Blanchon T. Boulanger N. Camus D. Cazorla C. Hansmann Y. Leparc-Goffart I. (2020). *Avis Relatif à L’inscription de L’encéphalite à Tiques sur la Liste des Maladies à Déclaration Obligatoire.* Paris: Haut conseil de Sant Publique. 1–22.

[B10] Blaskovic D. (1967). The public health importance of tick-borne encephalitis in Europe. *Bull. World Health Organ.* 36(Suppl.), 5–13. 5298542 PMC2476097

[B11] Bogovic P. Lotric-Furlan S. Strle F. (2010). What tick-borne encephalitis may look like: clinical signs and symptoms. *Travel Med. Infect. Dis.* 8 246–250. 10.1016/j.tmaid.2010.05.011 20970727

[B12] Botelho-Nevers E. Gagneux-Brunon A. Velay A. Guerbois-Galla M. Grard G. Bretagne C. (2019). Tick-Borne Encephalitis in Auvergne-Rhône-Alpes Region. France, 2017-2018. *Emerg. Infect. Dis.* 25 1944–1948. 10.3201/eid2510.181923 PMC6759258 31538929

[B13] Bournez L. Umhang G. Faure E. Boucher J. M. Boué F. Jourdain E. (2019). Exposure of wild ungulates to the usutu and tick-borne encephalitis viruses in France in 2009-2014: evidence of undetected flavivirus circulation a decade ago. *Viruses* 12:10. 10.3390/v12010010 31861683 PMC7019733

[B14] Bournez L. Umhang G. Moinet M. Richomme C. Demerson J.-M. Caillot C. (2020). Tick-Borne encephalitis virus: seasonal and annual variation of epidemiological parameters related to nymph-to-larva transmission and exposure of small mammals. *Pathogens.* 9:518. 10.3390/pathogens9070518 32605114 PMC7400523

[B15] Brabec M. Daniel M. Malý M. Danielová V. Kříž B. Kott I. (2017). Analysis of meteorological effects on the incidence of tick-borne encephalitis in the czech republic over a thirty-year period. *Virol. Res. Rev.* 2017 1–8.

[B16] Brockmann S. O. Oehme R. Buckenmaier T. Beer M. Jeffery-Smith A. Spannenkrebs M. (2018). A cluster of two human cases of tick-borne encephalitis (TBE) transmitted by unpasteurised goat milk and cheese in Germany. May 2016. *Euro. Surveill.* 23:17–0036. 10.2807/1560-7917.ES.2018.23.15.17-00336 PMC6836198 29667575

[B17] Caini S. Szomor K. Ferenczi E. Szekelyne Gaspar A. Csohan A. Krisztalovics K. (2012). Tick-borne encephalitis transmitted by unpasteurised cow milk in western hungary. september to October 2011. *Euro. Surveill.* 17: 20128. 22490310

[B18] Cannon R. M. (2001). Sense and sensitivity–designing surveys based on an imperfect test. *Prev. Vet. Med.* 49 141–163. 10.1016/s0167-5877(01)00184-2 11311950

[B19] Casati Pagani S. Frigerio Malossa S. Klaus C. Hoffmann D. Beretta O. Bomio-Pacciorini N. (2019). First detection of TBE virus in ticks and sero-reactivity in goats in a non-endemic region in the southern part of Switzerland (Canton of Ticino). *Ticks Tick Borne Dis.* 10 868–874. 10.1016/j.ttbdis.2019.04.006 31047827

[B20] Chernokhaeva L. L. Rogova Y. V. Kozlovskaya L. I. Romanova L. I. Osolodkin D. I. Vorovitch M. F. (2018). Experimental evaluation of the protective efficacy of tick-borne encephalitis (TBE) vaccines based on european and far-eastern TBEV strains in mice and in vitro. *Front. Microbiol* 9:1487. 10.3389/fmicb.2018.01487 30061869 PMC6054986

[B21] Chitimia-Dobler L. Lindau A. Oehme R. Bestehorn-Willmann M. Antwerpen M. Drehmann M. (2021). Tick-borne encephalitis vaccination protects from alimentary TBE infection: results from an alimentary outbreak. *Microorganisms* 9:889. 10.3390/microorganisms9050889 33919318 PMC8143337

[B22] Cisak E. Wójcik-Fatla A. Zaja̧c V. Sroka J. Buczek A. Dutkiewicz J. (2010). Prevalence of tick-borne encephalitis virus (TBEV) in samples of raw milk taken randomly from cows, goats and sheep in eastern Poland. *Ann. Agric. Environ. Med.* 17 283–286. 21186771

[B23] Csángó P. A. Blakstad E. Kirtz G. C. Pedersen J. E. Czettel B. (2004). Tick-borne encephalitis in southern Norway. *Emerg. Infect. Dis.* 10 533–534. 10.3201/eid1003.020734 15109431 PMC3322774

[B24] Daniel M. Danielová V. Kriz B. Kott I. (2004). An attempt to elucidate the increased incidence of tick-borne encephalitis and its spread to higher altitudes in the Czech Republic. *Int. J. Med. Microbiol.* 293(Suppl. 37), 55–62. 10.1016/s1433-1128(04)80009-3 15146985

[B25] Desgrandchamps D. Posfay-Barbe K. M. Schmitt H. J. (2019). “Chapter 12b – A: TBE in Switzerland and Liechtenstein,” in *The TBE Book*, 2nd Edn. eds Dobler G. Erber W. Bröker M. Schmitt H. J. (Singapore: Global Health Press), 342–347.

[B26] Deviatkin A. A. Kholodilov I. S. Vakulenko Y. A. Karganova G. G. Lukashev A. N. (2020). Tick-borne encephalitis virus: an emerging ancient zoonosis? *Viruses* 12:247. 10.3390/v12020247 32102228 PMC7077300

[B27] Dobler P. D. G. Erber W. Bröker M. Schmit P. D. H. J. (2019). *The TBE Book*, IInd Edn. Singapore: Global Health Press Pte Limited.

[B28] Dollat M. Bellanger A.-P. Millon L. Chirouze C. Lepiller Q. Marguet P. (2021). Knowledge and vaccination practices among family physicians in northeastern France regarding tick-borne encephalitis virus. *Ticks and Tick-borne Dis.* 12:101774. 10.1016/j.ttbdis.2021.101774 34175735

[B29] Donoso Mantke O Escadafal C. Niedrig M. Pfeffer M. Working Group for Tick-Borne Encephalitis Virus C. (2011). Tick-borne encephalitis in Europe, 2007 to 2009. *Euro. Surveill.* 16 19976. 10.2807/ese.16.39.19976-en 21968423

[B30] Dorko E. Hockicko J. Rimárová K. Bušová A. Popad’ák P. Popad’áková J. (2018). Milk outbreaks of tick-borne encephalitis in Slovakia, 2012-2016. *Eur. J. Public Health* 26(Suppl.), S47–S50. 10.21101/cejph.a5272 30817873

[B31] Driouich J. S. Moureau G. De Lamballerie X. Nougairède A. (2019). Reverse Genetics of RNA viruses: ISA-based approach to control viral population diversity without modifying virus phenotype. *Viruses* 11:666. 10.3390/v11070666 31330809 PMC6669666

[B32] ECDC. (2012). *Epidemiological Situation of tick-Borne Encephalitis in the European Union and European free trade association countries.* Stockholm: European Centre for Disease Prevention and Control.

[B33] ECDC. (2018). *Tick-Borne Encephalitis: Epidemiological Report for 2016.* Stockholm: European Centre for Disease Prevention and Control, 1–7.

[B34] Gondard M. Michelet L. Nisavanh A. Devillers E. Delannoy S. Fach P. (2018). Prevalence of tick-borne viruses in *Ixodes ricinus* assessed by high-throughput real-time PCR. *Pathog. Dis.* 2018:76. 10.1093/femspd/fty083 30423120

[B35] Gresikova M. (1958). Recovery of the tick-borne encephalitis virus from the blood and milk of subcutaneously infected sheep. *Acta. Virol.* 2 113–119. 13545072

[B36] Gresíková M. Sekeyová M. Stúpalová S. Necas S. (1975). Sheep milk-borne epidemic of tick-borne encephalitis in Slovakia. *Intervirology* 5 57–61. 10.1159/000149880 1237478

[B37] Gunnar H. Gunnar B. Erik E. Christer J. Bjørn L. Jan Erik R. (2009). Transport of Ticks by Migratory Passerine Birds to Norway. *J. Parasitol.* 95 1342–1351. 10.1645/GE-2146.1 19658452

[B38] Hansmann Y. Pierre Gut J. Remy V. Martinot M. Allard Witz M. Christmann D. (2006). Tick-borne encephalitis in eastern France. *Scand. J. Infect. Dis.* 38 520–526. 16798704 10.1080/00365540600585073

[B39] Hellenbrand W. Kreusch T. Böhmer M. M. Wagner-Wiening C. Dobler G. Wichmann O. (2019). Epidemiology of Tick-borne encephalitis (TBE) in Germany, 2001?2018. *Pathogens* 8:42. 10.3390/pathogens8020042 30934855 PMC6630332

[B40] Hennechart-Collette C. Martin-Latil S. Fraisse A. Perelle S. (2017). Comparison of three extraction methods to detect noroviruses in dairy products. *Food Microbiol.* 61 113–119. 10.1016/j.fm.2016.09.001 27697160

[B41] Herpe B. Schuffenecker I. Pillot J. Malvy D. Clouzeau B. Bui N. (2007). Tickborne encephalitis, southwestern France. *Emerg. Infect. Dis.* 13 1114–1116. 10.3201/eid1307.070041 18214196 PMC2878238

[B42] Holding M. Dowall S. D. Medlock J. M. Carter D. P. Pullan S. T. Lewis J. (2020). Tick-Borne Encephalitis Virus. United Kingdom. *Emerg. Infect. Dis.* 26 90–96. 10.3201/eid2601.191085 PMC6924911 31661056

[B43] Holzmann H. Aberle S. W. Stiasny K. Werner P. Mischak A. Zainer B. (2009). Tick-borne encephalitis from eating goat cheese in a mountain region of Austria. *Emerg. Infect. Dis.* 15 1671–1673. 10.3201/eid1510.090743 19861072 PMC2866415

[B44] Hudopisk N. Korva M. Janet E. Simetinger M. Grgič-Vitek M. Gubenšek J. (2013). Tick-borne encephalitis associated with consumption of raw goat milk. Slovenia, 2012. *Emerg. Infect. Dis.* 19 806–808. 10.3201/eid1905.121442 PMC3647507 23697658

[B45] Ilic M. Barbic L. Bogdanic M. Tabain I. Savic V. Kosanovic Licina M. L. (2020). Tick-borne encephalitis outbreak following raw goat milk consumption in a new micro-location. Croatia, June 2019. *Ticks Tick borne Dis.* 11:101513. 10.1016/j.ttbdis.2020.101513 32993933

[B46] Imhoff M. Hagedorn P. Schulze Y. Hellenbrand W. Pfeffer M. Niedrig M. (2015). Review: Sentinels of tick-borne encephalitis risk. *Ticks Tick Borne Dis.* 6 592–600. 10.1016/j.ttbdis.2015.05.001 26005107

[B47] Jaenson T. G. Hjertqvist M. Bergström T. Lundkvist A. (2012). Why is tick-borne encephalitis increasing? A review of the key factors causing the increasing incidence of human TBE in Sweden. *Parasit Vectors* 5:184. 10.1186/1756-3305-5-184 22937961 PMC3439267

[B48] Jahfari S. De Vries A. Rijks J. M. Van Gucht S. Vennema H. Sprong H. (2017). Tick-Borne encephalitis virus in ticks and roe deer, the Netherlands. *Emerg. Infect. Dis.* 23 1028–1030. 10.3201/eid2306.161247 28518024 PMC5443429

[B49] Kerlik J. Avdičová M. Štefkovičová M. Tarkovská V. Pántiková Valachová M. Molčányi T. (2018). Slovakia reports highest occurrence of alimentary tick-borne encephalitis in Europe: Analysis of tick-borne encephalitis outbreaks in Slovakia during 2007–2016. *Travel Med. Infect. Dis.* 26 37–42. 10.1016/j.tmaid.2018.07.001 30012472

[B50] Klaus C. Beer M. Saier R. Schau U. Moog U. Hoffmann B. (2012). Goats and sheep as sentinels for tick-borne encephalitis (TBE) virus – Epidemiological studies in areas endemic and non-endemic for TBE virus in Germany. *Ticks Tick-borne Dis.* 3 27–37. 10.1016/j.ttbdis.2011.09.011 22309856

[B51] Klaus C. Ziegler U. Hoffmann D. Press F. Fast C. Beer M. (2019). Tick-borne encephalitis virus (TBEV) antibodies in animal sera - occurrence in goat flocks in Germany, longevity and ability to recall immunological information after more than six years. *BMC Vet. Res.* 15:399. 10.1186/s12917-019-2157-5 31694666 PMC6836345

[B52] Klaus C. Ziegler U. Kalthoff D. Hoffmann B. Beer M. (2014). Tick-borne encephalitis virus (TBEV) – findings on cross reactivity and longevity of TBEV antibodies in animal sera. *BMC Vet. Res.* 10:78. 10.1186/1746-6148-10-78 24690234 PMC3978054

[B53] Kohlmaier B. Schweintzger N. A. Sagmeister M. G. Švendová V. Kohlfürst D. S. Sonnleitner A. (2021). Clinical characteristics of patients with tick-borne encephalitis (TBE): a european multicentre study from 2010 to 2017. *Microorganisms* 9:1420. 10.3390/microorganisms9071420 34209373 PMC8306415

[B54] Kubinski M. Beicht J. Gerlach T. Volz A. Sutter G. Rimmelzwaan G. F. (2020). Tick-borne encephalitis virus: a quest for better vaccines against a virus on the rise. *Vaccines* 8:451. 10.3390/vaccines8030451 32806696 PMC7564546

[B55] Labuda M. Kozuch O. Zuffová E. Elecková E. Hails R. S. Nuttall P. A. (1997). Tick-borne encephalitis virus transmission between ticks cofeeding on specific immune natural rodent hosts. *Virology* 235 138–143. 10.1006/viro.1997.8622 9300045

[B56] Lefort V. Longueville J.-E. Gascuel O. (2017). SMS: smart model selection in PhyML. *Mol. Biol Evol.* 34 2422–2424. 10.1093/molbev/msx149 28472384 PMC5850602

[B57] Li H. (2018). Minimap2: pairwise alignment for nucleotide sequences. *Bioinformatics* 34 3094–3100. 10.1093/bioinformatics/bty191 29750242 PMC6137996

[B58] Li H. Durbin R. (2009). Fast and accurate short read alignment with burrows-wheeler transform. *Bioinformatics* 25 1754–1760. 10.1093/bioinformatics/btp324 19451168 PMC2705234

[B59] Liebig K. Boelke M. Grund D. Schicht S. Bestehorn-Willmann M. Chitimia-Dobler L. (2021). The Stable Matching Problem in TBEV enzootic circulation: how important is the perfect tick-virus match? *Microorganisms* 9:196. 10.3390/microorganisms9010196 33477924 PMC7833397

[B60] Liebig K. Boelke M. Grund D. Schicht S. Springer A. Strube C. (2020). Tick populations from endemic and non-endemic areas in Germany show differential susceptibility to TBEV. *Sci. Rep.* 10:15478. 10.1038/s41598-020-71920-z 32968088 PMC7511395

[B61] Lindquist L. Vapalahti O. (2008). Tick-borne encephalitis. *Lancet* 371 1861–1871. 18514730 10.1016/S0140-6736(08)60800-4

[B62] Lukan M. Bullova E. Petko B. (2010). Climate warming and tick-borne encephalitis. Slovakia. *Emerg Infect. Dis.* 16 524–526. 10.3201/eid1603.081364 PMC3321998 20202437

[B63] Madeira F. Park Y. M. Lee J. Buso N. Gur T. Madhusoodanan N. (2019). The EMBL-EBI search and sequence analysis tools APIs in 2019. *Nucleic. Acids Res.* 47 W636–W641. 10.1093/nar/gkz268 30976793 PMC6602479

[B64] Markovinović L. Kosanović Ličina M. L. Tešić V. Vojvodić D. Vladušić Lucić I. Kniewald T. (2016). An outbreak of tick-borne encephalitis associated with raw goat milk and cheese consumption. Croatia, 2015. *Infection* 44 661–665. 10.1007/s15010-016-0917-8 27364148

[B65] Martello E. Mannelli A. Ragagli C. Ambrogi C. Selmi M. Ceballos L. A. (2014). Range expansion of Ixodes ricinus to higher altitude, and co-infestation of small rodents with Dermacentor marginatus in the Northern Apennines. Italy. *Ticks Tick Borne Dis.* 5 970–974. 10.1016/j.ttbdis.2014.07.021 25139531

[B66] Paulsen K. M. Stuen S. Das Neves C. G Suhel F. Gurung D. Soleng A. (2019). Tick-borne encephalitis virus in cows and unpasteurized cow milk from Norway. *Zoonoses Public Health* 66 216–222. 10.1111/zph.12554 30593734

[B67] Perez-Eid C. Hannoun C. Rodhain F. (1992). The Alsatian tick-borne encephalitis focus: presence of the virus among ticks and small mammals. *Eur. J. Epidemiol.* 8 178–186. 10.1007/BF00144797 1644133

[B68] Pérez-Eid C. (2007). *Les Tiques: Identification, Biologie, Importance Médicale et Vétérinaire., Monographies de Microbiologie.* Paris: TEC and DOC Lavoisier.

[B69] Peyrefitte C. N. Pastorino B. A. Bessaud M. Gravier P. Tock F. Couissinier-Paris P. (2005). Dengue type 3 virus, saint Martin, 2003-2004. *Emerg. Infect. Dis.* 11 757–761. 10.3201/eid1105.040959 15890134 PMC3320377

[B70] PROMED. (2020). *Tick-Borne Encephalitis – Germany (02). Archive Number: 20200907.7755195.* Boston, MA: Promed Post.

[B71] Quick J. Grubaugh N. D. Pullan S. T. Claro I. M. Smith A. D. Gangavarapu K. (2017). Multiplex PCR method for minion and illumina sequencing of zika and other virus genomes directly from clinical samples. *Nat. Protoc.* 12 1261–1276. 10.1038/nprot.2017.066 28538739 PMC5902022

[B72] Randolph S. E. (2010). To what extent has climate change contributed to the recent epidemiology of tick-borne diseases? *Vet. Parasitol.* 167 92–94. 10.1016/j.vetpar.2009.09.011 19833440

[B73] Randolph S. E. Miklisová D. Lysy J. Rogers D. J. Labuda M. (1999). Incidence from coincidence: patterns of tick infestations on rodents facilitate transmission of tick-borne encephalitis virus. *Parasitology* 118(Pt 2), 177–186. 10.1017/s0031182098003643 10028532

[B74] Reusken C. Boonstra M. Rugebregt S. Scherbeijn S. Chandler F. Avšič-Županc T. (2019). An evaluation of serological methods to diagnose tick-borne encephalitis from serum and cerebrospinal fluid. *J.Clin. Virol.* 120 78–83. 10.1016/j.jcv.2019.09.009 31590114

[B75] Rieille N. Klaus C. Hoffmann D. Péter O. Voordouw M. J. (2017). Goats as sentinel hosts for the detection of tick-borne encephalitis risk areas in the Canton of Valais, Switzerland. *BMC Vet. Res.* 13:217. 10.1186/s12917-017-1136-y 28693561 PMC5504567

[B76] Rónai Z. Egyed L. (2020). Survival of tick-borne encephalitis virus in goat cheese and milk. *Food Environ. Virol.* 12 264–268. 10.1007/s12560-020-09427-z 32388731

[B77] Rubel F. Brugger K. (2020). Tick-borne encephalitis incidence forecasts for Austria. Germany, and Switzerland. *Ticks Tick-borne Dis.* 11:101437. 10.1016/j.ttbdis.2020.101437 32723631

[B78] Rubel F. Brugger K. (2021). Operational TBE incidence forecasts for Austria. Germany, and Switzerland 2019-2021. *Ticks Tick Borne Dis.* 12:101579. 10.1016/j.ttbdis.2020.101579 33080518

[B79] Rushton J. O. Lecollinet S. Hubálek Z. Svobodová P. Lussy H. Nowotny N. (2013). Tick-borne encephalitis virus in horses. Austria, 2011. *Emerg. Infect. Dis.* 19 635–637. 10.3201/eid1904.121450 PMC3647421 23631894

[B80] Smura T. Tonteri E. Jääskeläinen A. Von Troil G. Kuivanen S. Huitu O. (2019). Recent establishment of tick-borne encephalitis foci with distinct viral lineages in the Helsinki area. Finland. *Emerg. Microbes Infect.* 8 675–683. 10.1080/22221751.2019.1612279 PMC6522972 31084456

[B81] Steffen R. Lautenschlager S. Fehr J. (2020). Travel restrictions and lockdown during the COVID-19 pandemic-impact on notified infectious diseases in Switzerland. *J. Travel Med.* 27 180. 10.1093/jtm/taaa180 33152761 PMC7543597

[B82] Süss J. (2011). Tick-borne encephalitis 2010: epidemiology, risk areas, and virus strains in Europe and Asia—An overview. *Ticks and Tick-borne Dis.* 2 2–15. 10.1016/j.ttbdis.2010.10.007 21771531

[B83] Tonteri E. Jokelainen P. Matala J. Pusenius J. Vapalahti O. (2016). Serological evidence of tick-borne encephalitis virus infection in moose and deer in Finland: sentinels for virus circulation. *Parasit. Vect.* 9:54. 10.1186/s13071-016-1335-6 26825371 PMC4733276

[B84] Ullrich A. Schranz M. Rexroth U. Hamouda O. Schaade L. Diercke M. (2021). Impact of the COVID-19 pandemic and associated non-pharmaceutical interventions on other notifiable infectious diseases in Germany: An analysis of national surveillance data during week 1–2016 – week 32–2020. *Lancet Reg. Health Eur.* 6:100103. 10.1016/j.lanepe.2021.100103 34557831 PMC8454829

[B85] Van Tongeren H. A. (1955). Encephalitis in Austria. IV. Excretion of virus by milk of the experimentally infected goat. *Arch. Gesamte Virusforsch.* 6 158–162. 10.1007/bf01247065 13259506

[B86] Velay A. Solis M. Kack-Kack W. Gantner P. Maquart M. Martinot M. (2018). A new hot spot for tick-borne encephalitis (TBE): a marked increase of TBE cases in France in 2016. *Ticks Tick Borne Dis.* 9 120–125. 10.1016/j.ttbdis.2017.09.015 28988602

[B87] Waldenström J. Lundkvist A. Falk K. I. Garpmo U. Bergström S. Lindegren G. (2007). Migrating birds and tickborne encephalitis virus. *Emerg. Infect. Dis.* 13 1215–1218. 10.3201/eid1308.061416 17953095 PMC2828075

[B88] Wallenhammar A. Lindqvist R. Asghar N. Gunaltay S. Fredlund H. Davidsson Å (2020). Revealing new tick-borne encephalitis virus foci by screening antibodies in sheep milk. *Parasit. Vect.* 13:185. 10.1186/s13071-020-04030-4 32268924 PMC7140392

